# HIPdb: A Database of Experimentally Validated HIV Inhibiting Peptides

**DOI:** 10.1371/journal.pone.0054908

**Published:** 2013-01-24

**Authors:** Abid Qureshi, Nishant Thakur, Manoj Kumar

**Affiliations:** Bioinformatics Centre, Institute of Microbial Technology, Council of Scientific and Industrial Research, Chandigarh, India; Aberystwyth University, United Kingdom

## Abstract

**Background:**

Besides antiretroviral drugs, peptides have also demonstrated potential to inhibit the Human immunodeficiency virus (HIV). For example, T20 has been discovered to effectively block the HIV entry and was approved by the FDA as a novel anti-HIV peptide (AHP). We have collated all experimental information on AHPs at a single platform.

**Descriptions:**

HIPdb is a manually curated database of experimentally verified HIV inhibiting peptides targeting various steps or proteins involved in the life cycle of HIV e.g. fusion, integration, reverse transcription etc. This database provides experimental information of 981 peptides. These are of varying length obtained from natural as well as synthetic sources and tested on different cell lines. Important fields included are peptide sequence, length, source, target, cell line, inhibition/IC_50_, assay and reference. The database provides user friendly browse, search, sort and filter options. It also contains useful services like BLAST and ‘Map’ for alignment with user provided sequences. In addition, predicted structure and physicochemical properties of the peptides are also included.

**Conclusion:**

HIPdb database is freely available at http://crdd.osdd.net/servers/hipdb. Comprehensive information of this database will be helpful in selecting/designing effective anti-HIV peptides. Thus it may prove a useful resource to researchers for peptide based therapeutics development.

## Introduction

Human Immunodeficiency Virus remains one of global public health concern due to the lack of vaccines and effective drugs to completely cure AIDS [1], [2], [3]. As per the World Health Organization reports, by the end of 2011, more than 1.8 million people all over the world have died because of this infection. Currently, an estimated 34 million people are infected with HIV worldwide (http://www.unaids.org/en/media/unaids/contentassets/documents/unaidspublication/2011/JC2216_WorldAIDSday_report_2011_en.pdf). Existing anti HIV drugs have been shown to cause toxic side effects and selection of resistant mutants and focus on only reverse transcriptase and protease enzymes of the virus [4]. However, various steps in the life cycle of HIV could be promising targets for intervention. Anti-HIV peptides (AHP) have shown budding potential to exploit these targets because of peptides’ specificity and diversity [5].

The first AHP, SJ-2176, inhibited the entry of the virus in the host cell and was derived from the C-terminal heptad repeat of gp41 [6]. Many researchers have used different types of peptides from both natural and synthetic sources to intercept different functions involved in the life cycle of HIV like fusion with the host cell [7], [8], reverse transcription [9], [10], or proteins like integrase [11], [12], protease [13], [14], vpr [15] etc. Moreover, peptides have many advantages like low toxicity, rapid elimination, less side effects etc. which sometimes makes them preferable over other compounds [5]. Further, peptide libraries enable to conveniently choose peptide ligands for any target that can act as lead for further improvement by informatics approaches and chemical conjugations [16].

Currently many synthetically designed peptides have reached clinical trials as antimicrobial compounds [17]. The peptide DP178 (T20, Enfuvirtide & Fuzeon) is the first anti-HIV peptide approved by the FDA [18]. Similarly, another peptide inhibitor, Sifuvirtide (SFT) has shown potent anti-HIV activity and pharmacokinetic profiles and is under phase-II clinical trial [19]. After Enfuvirtide, a number of design strategies have been exploited to synthesize new peptide inhibitors with enhanced stability, bioavailability and potency [20]. This further emphasize the peptides’ potential as alternative drugs for the treatment of HIV [21].

Currently there is no dedicated database archiving the experimental data on HIV inhibiting peptides. Of the available few antimicrobial peptide databases, only APD2 [22] and CAMP [23] databases reported limited 53 and 56 AHP entries respectively. Recently we have published a web server “AVPpred” for prediction of highly effective antiviral peptides [24] in which we have employed 209 AHP from our collection. We have incorporated all these earlier reported AHP in our newly developed AHP database.

## Materials and Methods

### Data Collection

The literature in Pubmed database was searched using three keywords including virus name, peptide and inhibition action. i) HIV OR Human immunodeficiency virus; ii) Peptide OR peptides; iii) Inhibit* OR block*. ‘Inhibit*’ is used to include various combinations of ‘inhibition’ and ‘inhibiting’ etc. Following Pubmed search query was used to retrieve literature- ((((HIV) OR Human immunodeficiency virus)) AND ((peptide) OR peptides)) AND ((inhibit*) OR block*).

Above Pubmed query returned 8216 articles in the Full-text search as on 12-07-2012. We screened many articles, however majority of them were not furnishing the desired data. This could be due to the fact that the above keywords are quite frequently used in the research articles. Therefore, we limited our query in the title/abstract fields to retrieve 2575 articles using the advanced search option of Pubmed. We again screened these articles based on abstracts or full paper to shortlist around 400 potential articles having the required data. These articles were manually examined in detail to fish out the desired data.

Simultaneously, following criteria were applied to filter out the articles. Papers those were limited in giving information only on predicted peptides or design, peptide structural studies, peptide analogues, dendrimeric peptides, complex peptide conjugates, peptides used in combination with drugs, peptide lacking sequence or experimental efficacy were removed. Review articles as well as non-English articles were also not considered. Emphasis was laid on to articles having experimentally validated peptides and covering all or most of the selected HIPdb fields. Besides, we have also searched these keywords in the PatentLens database and included data from three relevant patents. After filtering out the above articles, from remaining 112 full-length research articles where methods, results and conclusions are fully reported were finally used to collect 981 peptides experimentally tested for HIV inhibiting activity. Further modified peptides (87) and peptides with very low/nil activity (179) are provided separately.

### Data Organization

HIPdb currently archives the following fields extracted from the literature -

*Sequence*: All peptide sequences are formatted in standard one letter amino acid notation.

*Nomenclature:* Peptide designation from the respective study.

*Source*: The origin of the peptide including the organism name/parent protein.

*Cell line*: Cells on which the experiments are performed.

*Inhibition/IC_50_*: The inhibitory activity of the peptide, either qualitative or quantitative as discussed in the respective research article. IC_50_ (The half maximal inhibitory concentration) is a measure of the effectiveness of the peptide in inhibiting the virus.

*Unit*: Unit(s) of quantitative information provided in the preceding column.

*Target*: The protein/molecular process targeted by the peptide to inhibit HIV.

*Assay*: Experimental validation method.

*Reference*: References are given as Pubmed ID’s with external links pointing to the abstract of the article.

**Figure 1:**
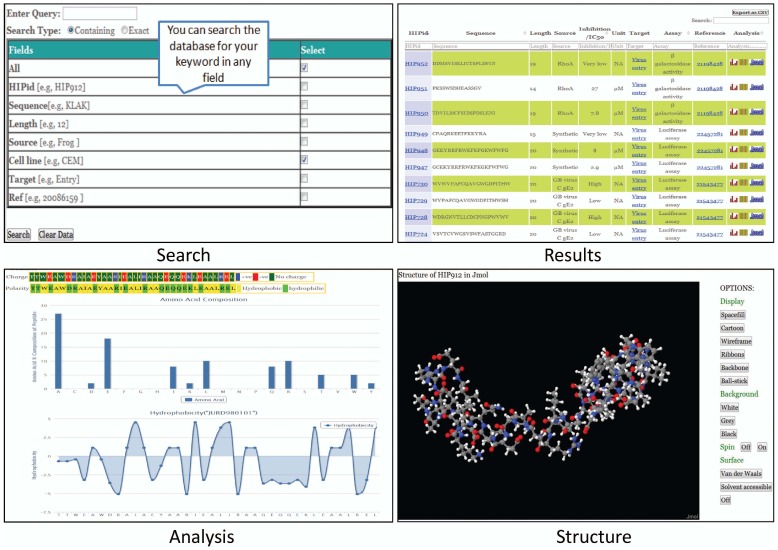
User interfaces in HIPdb.

In addition to the above information, other fields like physicochemical properties, blast results and structure of the peptide are also included in the respective entry. Physicochemical properties shown are charge, polarity, composition, hydrophobicity and secondary structure preference. The values used for calculating these properties were retrieved from the AAindex database [25]. Blast result shows the similar peptides reported in the database. Structures of the peptides were predicted using the PepStr algorithm [26]. Structures are displayed in Jmol applet. To view the structures java plugin should be installed in the browser, and javascript to be enabled. Database overview is given in **Figure 1**.

### Web Interface and Application

HIPdb has been created using the open source LAMP (Linux-Apache-MySQL-PHP) solution stack. The whole software system runs on IBM SAS ×3800 machine under Red Hat Enterprise Linux 5 environment using Apache httpd server.

### Data Retrieval

Users can query the database in a number of ways using browse, search and advance search facility. User can browse the database using five different categories viz. i) peptide source, ii) cell line, iii) target, iv) assay and v) author. Each of the categories is further classified to retrieve specific information. In the search page user can search the required information in the whole database or in the six designated fields. Radio buttons in the search page helps in finding entries having partial or exact keywords. The search output has the option to sort the data by clicking on columns’ title (**Figure 1**). Further user can filter the data by entering the desired keyword in the designated field. Multiple filtering/sorting can be accomplished by selecting columns & entering keywords in different fields one after another. The users also have the option to export their search results as a csv file. Further the advanced search page allows for more ﬂexible queries using logical operators (AND, OR). These options enable the user to readily find the appropriate data.

### Tools

HIPdb allows the users to take advantage of useful tools like HIPdb Map and HIPdb Blast. In map interface user can paste the peptide/protein sequence. Results will show mapping of peptide entries from our database on the user provided sequence. It helps the users to know that how many peptides have been reported earlier along with their positions on the sequence. Clicking on any result entry, user will get complete information available for that peptide in the HIPdb. Additionally, Blast tool allows alignment of a user provided peptide sequence against the sequences available in our database.

### Data Submission

Researchers working on HIV inhibitory peptides may submit their experimental data using the update page in the HIPdb. Submitted information will be included in the database update after ascertaining its authenticity.

## Results

HIPdb contains manually curated entries of 981 peptides checked for anti HIV activity. It also has information of 87 modified peptides which were having non natural amino acids or some attached chemical moiety. This database has entries of peptide targeting different steps involved in the pathogenesis of HIV-1. But majority of these were aimed at attachment, integrase, reverse transcriptase, multimerization and protease. The peptides that target surface interactions between the virus and the host cell are divided into (a) virus entry inhibitors which prevent binding of the virus envelope proteins to the CD4 or chemokine co-receptors and (b) fusion inhibitors which target interaction after productive binding to the cellular receptors.

**Figure 2:**
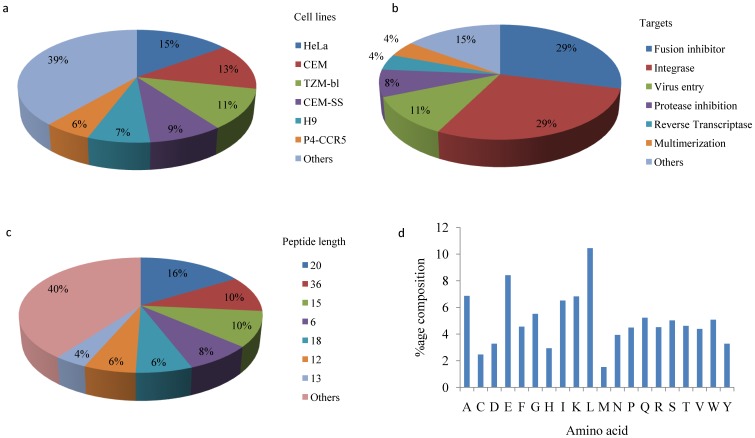
HIPdb statistics: a) Targets, b) Cell lines, c) Peptide length, d) Amino acid composition.

The peptides have been tested in 35 different cell lines but HeLa, CEM, and TZM-bl were more frequently used. Amino acids length of most of the peptides in our collection were in the range of 16–20 (28%) or 34 to 38 (17%) residues. However, peptides of length 20, 36 and 15 residues were commonly used. After calculating the amino acid composition of the peptides in HIPdb we found that residues like Leu, Glu and Ala, Lys, Ile and Gly were more frequent while Met, Cys, Tyr, His and Asp were least frequent as shown in **Figure 2**. To see the preference of amino acid residues, we have compared the amino acid composition of HIPdb and APD2 with Uniprot. We found that in HIPdb Trp was 4 fold enriched, while in APD2 Cys was 5.6 fold higher compared to uniprot (**Figure S1**). The efficacy of the peptides was tested through 56 different assays but Cell fusion assay, ELISA, RT production and Dot blot assays were used in most of the cases. Further in our database it was observed that the cell line CEM-174 was the most frequently used (considering least activity entries also) which is consistent with the fact that HIV-1 is able to replicate more robustly in this T-cell/B-cell hybrid line, CEM 174 [27].

The modified peptides were usually having D-Lys, D-Ile, D-Leu, D-Trp, D-Pro, D-Tyr, acetyl, benzyl, amide, amine, acetamidomethyl, S-ethyl, tetrahydro-isoquinoline-3-carboxylic acid, biotinyl, isobutyroyl, palmitoyl and phenylglycine moieties attached with them. Natural peptides have a short in vivo activity due to low bioavailability and proteolysis. Sometimes modified peptides are used to improve on these limitations, the so called peptidomimetics. However, several cases have failed to reproduce the efficacy of the original peptide [28].

## Discussion

There is a limited availability of therapeutic molecules for many viral infections [29]. Antiviral peptide therapeutics has the potential in combating viral infections [30], [31]. Particularly during the past decade there has been a considerable focus on HIV therapy and a lot of peptides have been tested against this pathogen. Jin et al. have used small peptides that can inhibit gp120/41-mediated cell fusion with an IC_50_ (the 50% inhibitory concentration) of 1–6 µM [8]. Gleenberg et al. has used two sequential peptides derived from vpr that inhibited both reverse transcriptase as well as integrase enzymes of the virus with IC_50_ values of 0.22–2 µM [10]. Similarly Li and co workers have identified a peptide that can inhibit the integrase enzyme of HIV with IC_50_ value of 2.7 µM [11]. Also, Schramm et al. have used peptides that are able to inhibit the HIV protease activity with IC_50_ values in the range of µM. Their best performing peptide achieved an IC_50_ of less than 1 µM [14]. These observations indicate that peptides were able to prevent the different processes of the virus mediated by these proteins.

The best inhibitory peptides could then be used as starting point for the design of more active molecules targeting the key steps involved in virus attachment, fusion and replication etc. Also, peptides which mimic the attachment sites of HIV proteins for their host cell receptors may be potential immunogen candidates for new vaccine strategies [5], [32].

The peptides collected in HIPdb were mostly from natural sources (75%) with majority from the E2 (GBV-C), gp41, reverse transcriptase and integrase proteins. Further around 10% of the total peptides entries were derived from phage display. Many peptides that have the ability to structurally mimic important substrates are actually derived from HIV itself, e.g., peptides derived from vpr have been found to be inhibitory to reverse transcriptase and integrase [33] while peptides corresponding to the C-terminal heptad repeat of HIV-1 gp41 are potent inhibitors of HIV-1 entry into cells [34]. Efficacy is given as IC_50_/inhibition percent. Quantitative information of efficacy is provided for most cases (614 entries) whereas in rest of the entries (367) a qualitative scale has been used as per the original reference with high (106 peptides), medium (86) and low (175) tags.

Despite immense potential, there is no HIV specific antiviral peptide database available. Although general antimicrobial databases exist on the web but they are having more entries for antibacterial peptides and very few entries against viruses, particularly HIV. For example out of 1228 APD2 entries only 53 are against HIV [22]. Similarly in CAMP database 2766 entries only 56 are anti HIV peptides [23]. Recently we published AVPpred web server for predicting highly effective antiviral peptides. It also provides collection of antiviral peptides with 209 peptides corresponding to HIV but the information is limited to sequences only [24]. While our database has many folds more anti-HIV peptides entries.

Further HIPdb provides unique data on experimentally validated HIV inhibitory peptides at a single platform. Compared to other antimicrobial peptide databases, it provides information of cell line, efficacy, target and assay which are not found in the former. In addition, tools to calculate some of the properties known to influence antiviral activity and structure of the peptides are also included. The data can be used to select or design better peptides for improved virus inhibition and this would be useful in the peptide based HIV therapeutics development.

### Limitations and Future Prospects

As increasing number of articles are being published in the area of HIV inhibitory peptides. Therefore, in future our priority would be to update the existing data. We will include information for other strains/types of HIV, once appropriate data is available.

### Availability and Requirements

This database is freely accessible on the web via the url http://crdd.osdd.net/servers/hipdb. Note that to view the structures and graphical applications java plugin as well as javascript should be enabled in the browser.

## Supporting Information

Figure S1 **Comparison of amino acid compositions of HIPdb, APD2 and Uniprot databases.**
(TIF)
