# Supplementary figures and images for: HIPdb: A Database of Experimentally Validated HIV Inhibiting Peptides
Source: PLoS One. 2013 Jan 24;8(1):e54908. doi: 10.1371/journal.pone.0054908 (PMC3554673; doi:10.1371/journal.pone.0054908)

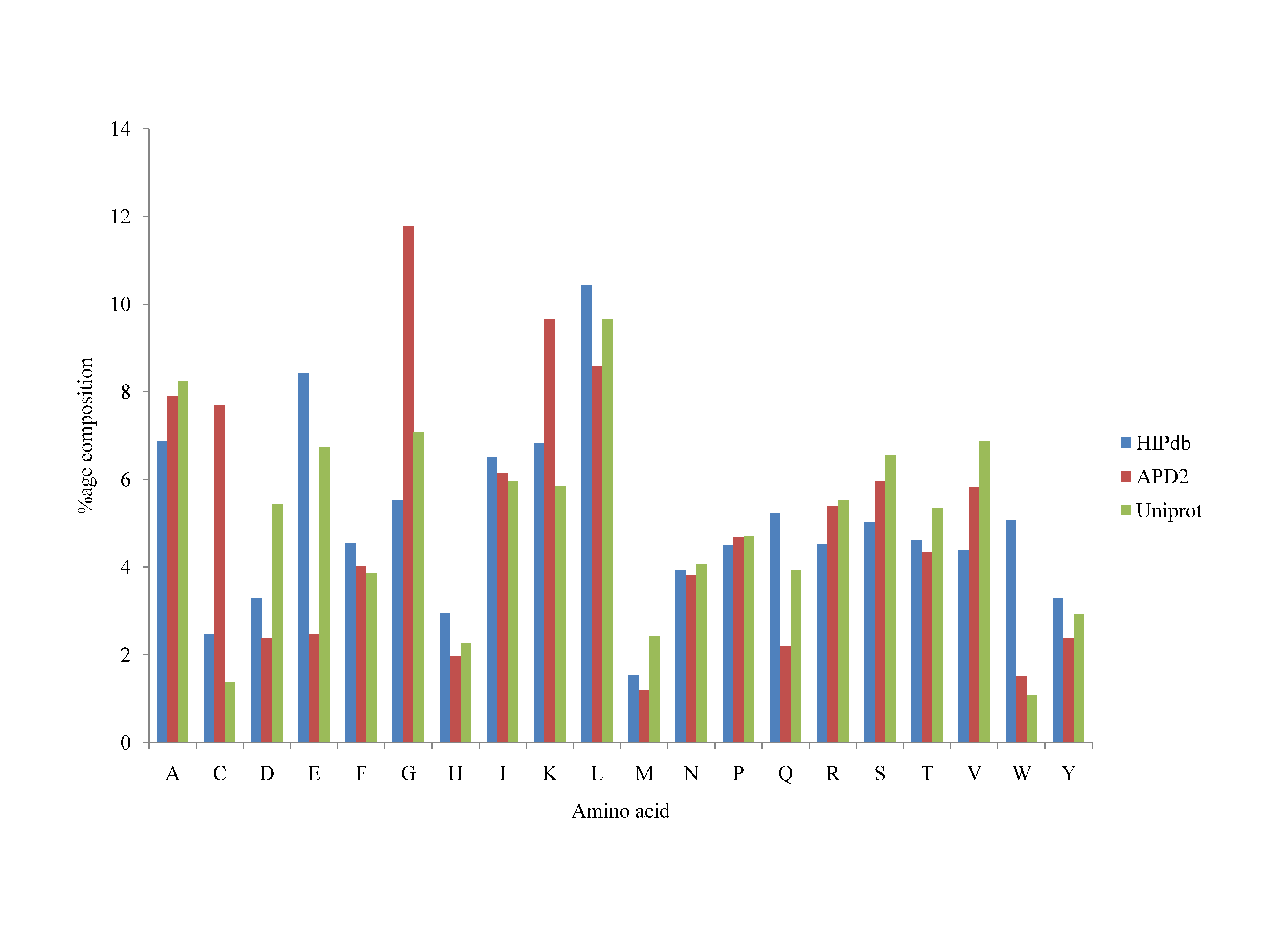

Supplement: Figure S1 — Comparison of amino acid compositions of HIPdb, APD2 and Uniprot databases. (TIF) [file pone.0054908.s001.tif]
